# Is Atrial Fibrillation a Risk Factor for Gastroesophageal Reflux Disease Occurrence?

**DOI:** 10.1097/MD.0000000000001921

**Published:** 2015-10-30

**Authors:** Jae Jin Hwang, Dong Ho Lee, Hyuk Yoon, Cheol Min Shin, Young Soo Park, Nayoung Kim

**Affiliations:** From the Department of Internal medicine, Seoul National University College of Medicine, Seoul National University Bundang Hospital, Seongnam, South Korea.

## Abstract

Recent studies have reported an association between gastroesophageal reflux disease (GERD) and atrial fibrillation (AF). The objective of the present study was to evaluate whether AF is one of the risk factors for GERD occurrence.

In this hospital-based, retrospective, case–control study, the patients were classified into 2 groups. The patients diagnosed with new AF were assigned to the AF group (n = 1612); those diagnosed without AF and GERD were assigned to the control group (n = 1612). The subjects in the control group were selected from outpatients of total healthcare center without a history of AF or GERD, and matched for age and gender. We evaluated the incidence of GERD and risk factors for GERD occurrence between the 2 groups.

The number of patients experiencing occurrence of GERD during the follow-up period was significantly higher in the AF group than those in the control group, respectively (129 patients vs 98 subjects, *P* = 0.037). The incidence of GERD was significantly higher in the AF group than in the control group by Kaplan–Meier analysis with log-rank test (*P* = 0.008). The AF group's adjusted hazard ratio of GERD occurrence against that of the control group was 1.37 (95% confidence interval [CI]: 1.16–1.57; *P* = 0.009) according to Cox's proportional hazard model.

The presence of AF appears to increase the incidence of GERD and may be considered a risk factor for the development of GERD. Further, large prospective and cohort studies will be required to better establish the correlation of GERD with AF.

## INTRODUCTION

Gastroesophageal reflux disease (GERD) is common gastrointestinal condition marked by heartburn and acid regurgitation. GERD is characterized by the movement of acidic gastric contents up into the esophagus, with or without esophageal mucosal damage.^1^ There have been several studies on the multifocal pathogenesis of GERD. Transient lower esophageal sphincter (LES) relaxation is considered to be the predominant mechanism by which gastric contents enter into the esophagus. Reduced esophageal motility is another, and manifests as an incapacity for full removal of acidic gastric contents from the esophagus. Other factors contributing to the pathophysiology of GERD include impaired mucosal defensive factors, delayed gastric emptying, ineffective esophageal acid and bolus clearance, and impairment of the esophagogastric junction.^2^ GERD has been increasingly correlated with obesity,^3^ metabolic syndrome,^4^ sleep apnea,^5^ aging, and, in developed countries, reduced incidence of *Helicobacter pylori* infection.^6^ With regard to lifestyle, a full stomach reduces LES tone,^7^ and obesity elevates the gastroesophageal pressure gradient.^3^ Hiatal hernia and LES relaxation are observed frequently in the elderly.^8^

Atrial fibrillation (AF) is a common arrhythmia generally known to be an age-dependent and progressive disease. AF prevalence increases from 0.5% at 50 to 59 years to 9.0% at 80 to 90 years.^9^ In the results of US and European community-based cohort studies, the lifetime risk of AF was 22% to 26% in men and 22% to 23% in women by 80 years.^10,11^ Because AF can impair quality of life by limiting exercise capacity and lead to coronary artery disease, stroke, heart failure, and even death, understanding its mechanisms of development and devising effective treatments is important. Such mechanisms, however, still are not entirely understood. The predominant initiatory mechanism is considered to be electrical triggers occurring around the myocardium and pulmonary vein. In 10% to 15% of cases, lone AF, characterized by the absence of co-morbidities, occurs.^10^ Some clinical studies have demonstrated that the frequency of AF episodes is significantly reduced and the symptoms of AF improved in patients with concomitant GERD after treatment with acid suppressive therapy. Meanwhile, new AF risk factors have been reported; these include metabolic syndrome,^12^ sleep apnea,^13^ alcohol use,^14^ systemic inflammation,^15^ and specific genetic mutations.^16,17^ The predisposing factors for AF overlap with those for GERD. It is not clear whether this overlap represents a strong correlation between GERD and AF or is just coincidental. In any case, the sharing of predisposing factors as well as the adjacent anatomical positioning and nerve innervations between the esophagus and atria suggest that a correlation between GERD and AF indeed exists. However, this remains controversial. Although several studies have reported that GERD may be one of the risk factors for AF occurrence, but there was only a study that AF may be one of the risk factors for GERD occurrence. The objective of the present study was to evaluate whether AF is one of the risk factors for GERD occurrence.

## METHODS

### Subject Selection

The medical records of patients diagnosed with AF at Seoul National University Bundang Hospital between January 1, 2011 and December 31, 2013 were retrospectively reviewed. The patients selected for the study met the following inclusion criteria: age over 18 years, patients who were diagnosed with new AF according to the *International Classification of Diseases, Ninth Revision* (*ICD-9*), patients who were diagnosed with nonvalvular AF, patients who were normal in the previous Esophagogastroduodenoscopy (EGD). The exclusion criteria were as follows: age below 18 years, previous diagnosis of AF or GERD according to the *ICD-9*, patients who were diagnosed with valvular AF, patients who were diagnosed with other gastrointestinal diseases (peptic ulcer, achalasia, gastric cancer, etc.). A control group was selected from outpatients without a history of AF or GERD at Seoul National University Bundang Hospital Total Health care center in the study periods. There may be a significant bias between patients who were diagnosed with AF and control group. Therefore, we used a 1:1 matching procedure on the computer, which eliminated biases as much as possible, to match all the patients by age and gender. The study protocol was approved by the Ethics Committee at Seoul National University Bundang Hospital (IRB Number: B-1406-256-110).

### Diagnosis of AF and GERD

AF was diagnosed by electrocardiography and 24 hr Holter monitoring testing. The diagnosis of AF using electrocardiography and 24 hr Holter monitoring testing are generally used, the diagnostic methods were applied in the similar studies.^18,19^ GERD was diagnosed by the presence of typical symptom (heartburn or the reflux of stomach contents cause troublesome symptoms and/or complications) and the positive results for 1 or more of 2 methods (the presence of the mucosal break in the EGD, ambulatory esophageal pH monitoring test). Heartburn is defined troublesome if mild symptoms occur 2 or more days a week, or moderate to severe symptoms occur more than 1 day a week.^20^ The diagnosis of GERD using these diagnostic methods are also generally used, the diagnostic methods were applied in the previous studies.^21,22^

### Study Protocol

The present study was a hospital-based, retrospective, case–control study. All the medical records of patients and control subjects were retrospectively reviewed. The patients were classified into 2 groups. Those diagnosed with new AF were assigned to the AF group; those diagnosed without AF and GERD were assigned to the control group. Additionally, demographic information (age, gender), co-morbidities (pre-existing *ICD-9* diagnoses of hypertension, coronary artery disease, diabetes mellitus, chronic obstructive pulmonary disease, dyslipidemia, congestive heart failure, ischemic stroke, thyrotoxicosis) arising more than 6 months postdiagnosis, history of drug use (calcium channel blocker, statin, warfarin, β-blocker, angiotensin receptor blocker, angiotensin-converting enzyme inhibitor, anti-diabetic agent, warfarin) occurring for more than 1 month postdiagnosis, other confounding factors (smoking, alcohol use, body mass index [BMI]) and occurrence of new-onset GERD were noted.

### Statistical Analysis

All statistical analysis was done using the Predictive Analytics Software (PASW) 20.0 version for Windows package (SPSS, Inc., IBM, Chicago, IL). The mean ± standard deviation was calculated for the quantitative variables. Student *t* test was used to evaluate continuous variables, and the chi-squared test and Fisher's exact test were used to assess noncontinuous variables. Kaplan–Meier analysis with log-rank test was employed to compare new-onset GERD occurrence between the AF and control groups. Cox's proportional hazard model was used to calculate the hazard ratio (HR) as corrected for smoking status, alcohol use, BMI, co-morbidities, and history of drug use for each group. A *P* value of <0.05 was defined as having clinical significance.

## RESULTS

### Occurrence of GERD

Between 2011 and 2013, a total of 1612 patients with newly diagnosed AF were identified from the retrospective review of the medical records and assigned to the AF group. Another 1612 control subjects, matched for age and gender were assigned to the control group.

Table 1 shows the occurrence of new-onset GERD for the 2 groups. In the AF group, 129 patients (8.0%) diagnosed with new-onset GERD during the follow-up period, while in the control group, 98 subjects (6.0%) diagnosed with new-onset GERD (*P* = 0.037, Table 1). The number of patients experiencing occurrence of new-onset GERD during the follow-up period was significantly higher in the AF group than those in the control group, respectively (*P* = 0.037, Table 1).

**TABLE 1:**
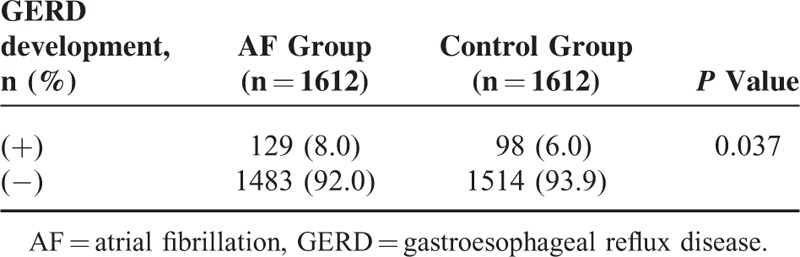
Occurrence of New-Onset GERD for the AF and Control Group

### Characteristics of Subjects for New-Onset GERD

The characteristics of the new-onset GERD patients in AF and control group are provided in Table 2. The average ages of the new-onset GERD patients in AF and control group were 68.34 ± 10.60 and 68.42 ± 11.55 years (*P* = 0.892). The overall follow-up period of the new-onset GERD patients in AF and control group were 40.18 ± 37.40 and 44.54 ± 33.49 months, respectively (*P* = 0.320). There were no differences in the gender distribution, smoking status, alcohol use, BMI, co-morbidities, and history of drug use between the 2 groups (*P* > 0.05).

**TABLE 2:**
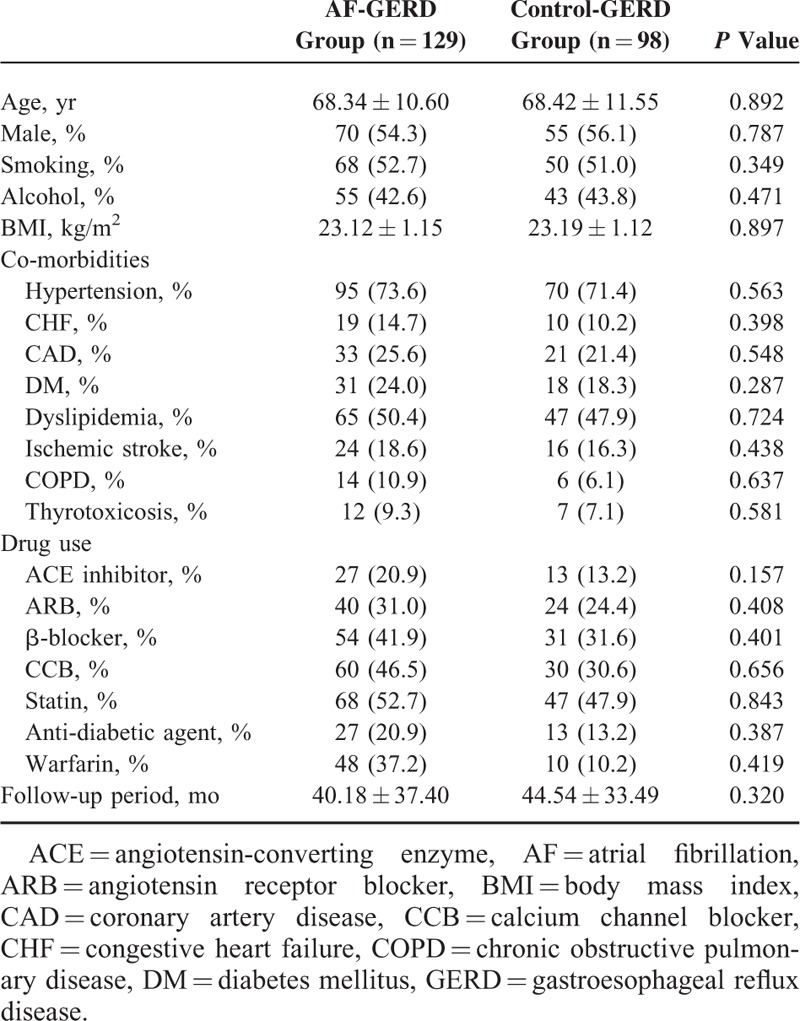
Baseline Characteristics of the New-Onset GERD Patients in AF and Control Group

### Incidence of New-Onset GERD

**FIGURE 1:**
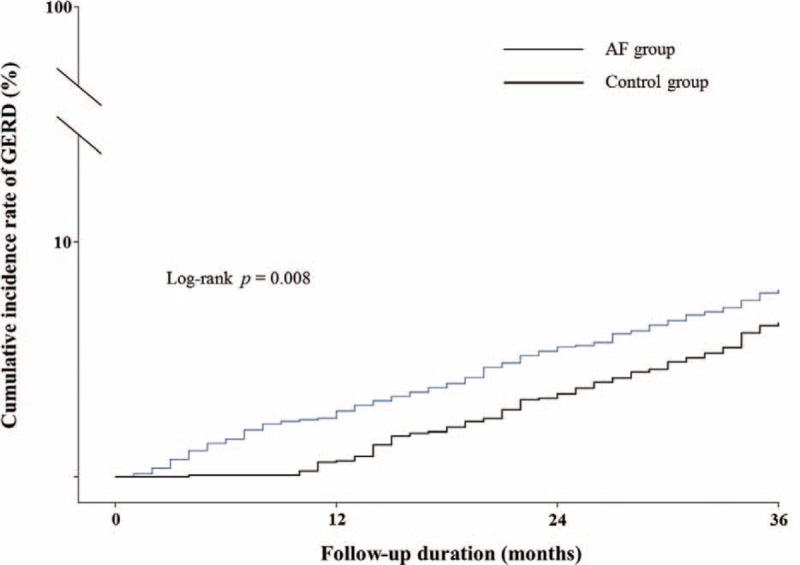
Kaplan–Meier curves of the cumulative incidence rate of GERD in the both groups. AF = atrial fibrillation, GERD = gastroesophageal reflux disease.

Figure 1 shows the Kaplan–Meier curves of the cumulative incidence rate of GERD in the both groups. Correspondingly, the incidence of GERD, by Kaplan–Meier analysis with log-rank test, was significantly higher in the AF group than in the control group (*P* = 0.008) (Fig. 1).

### Risk Factor for Occurrence of New-Onset GERD

The HR of new-onset GERD according to AF presence is provided in Table 3. The AF group's adjusted HR of GERD occurrence against that of the control group was 1.37 (95% confidence interval [CI]: 1.16–1.57; *P* = 0.009) according to Cox's proportional hazard model (Table 3). In this study, there was no statistically significant risk factor influencing for occurrence of new-onset GERD, except presence of AF.

**TABLE 3:**
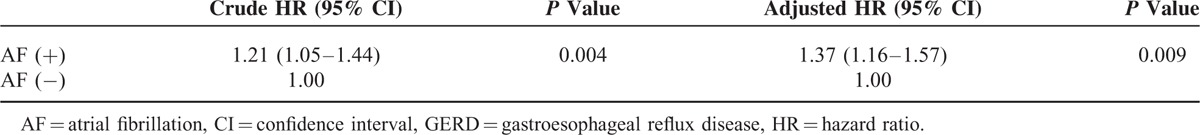
Hazard Ratio of New-Onset GERD According to Atrial Fibrillation Presence

## DISCUSSION

In this study, the incidence of GERD was higher in patient with AF than those without AF with statistically significant difference. These results suggested that AF would be one of the risk factors for GERD occurrence.

Several studies reported the correlation between GERD and AF. The first, Tougas et al^23^ employed ambulatory monitoring to determine the effects of esophageal stimulation on heart rate, reporting that esophageal stimulation, by the same mechanism as in AF initiation, increases the efferent vagal nerve activity. Weigl et al^24^ in a study on 89 GERD patients, identified 18 with lone AF, among whom the therapeutic effects of proton pump inhibitors (PPIs) were confirmed in 14 (78%). Gerson et al^25^ reported coincidence of acid reflux with paroxysms of AF and suppression of both acid reflux and AF paroxysms by PPI therapy on esophageal pH and simultaneous Holter monitoring testing. Cuomo et al^26^ found that for a number of dysrhythmia patients, autonomic reflux was induced due to esophageal acid reflux but that acid suppression improved the gastric and cardiac symptoms. Kunz et al,^27^ having reviewed a healthcare system database, were able to demonstrate a strong correlation between GERD and AF: specifically, after correction for other risk factors, the presence of GERD increased the relative risk for AF. Shimazu et al^28^ conducted a multicenter questionnaire survey on 188 subjects treated for GERD as outpatients, and identified AF as an independent risk factor for GERD. Gillnov and Rice^29^ reported on cases of hiatal hernia wherein patients underwent successful Nissen fundoplication that relieved their GERD symptoms, which improvement effected a positive heart-rhythm change from paroxysmal AF to a normal sinus rhythm. Contrarily, however, Bunch et al^21^ reported, on the basis of a large retrospective study that included a survey of 5288 residents of Olmsted County, MN, can be treated as no correlation between GERD and AF (HR: 0.81, 95% CI: 0.68–0.96, *P* = 0.014) after adjustment for other risk factors. The patients with a higher GERD frequency had a slightly higher AF risk; those with esophagitis were more likely to incur AF (HR: 1.94, 95% CI: 1.35–2.78, *P* < 0.001).^21^ Our study showed that patients with AF had significantly higher incidence of GERD than those without AF. These results suggested that AF may play a role in determining the risk of occurring GERD.

The potential common mechanism between GERD and AF remained unclear, though several case reports and retrospective studies have put forward 2 possible hypotheses: chronic local inflammation and autonomic over-stimulation.^21–29^

Inflammation of the atrium might be related to the pathogenesis of GERD or AF, particularly as the left atrium is in contact with the lower esophagus. Cummings et al^30^ reported a mean esophagus-to-left-atrium distance of 4.4 ± 1.2 mm. Atrial inflammatory reaction related to chronic AF theoretically confers a GERD-initiation mechanism via the adjacent anatomical association between the esophagus and the atria.^31^ In fact, it is known that inflammatory factors including oxidative stress, leukocytes, and cytokines such as interleukin (IL)-6 and IL-8 might play an important role in the occurrence of GERD. Among them, oxidative stress and cytokines (IL-6 and IL-8) are known also to play an important role in AF initiation.^32,33^ But the potential role of inflammatory factors such as oxidative stress and cytokines in the pathophysiology is unclear.

The receptors that over-stimulate the parasympathetic system are affected by acidic gastric contents or local mucosal inflammation in the esophagus via reflux loops involving the brain, similarly to bolus mechanism.^25,34^ Local inflammation of the esophageal mucosa induces afferent and efferent reflux mechanisms with involvement of the cerebral representation of the cardiac rhythm, thus leading to secondary stimulation of the vagal nerve.^34^ Over-stimulation of the vagal nerve effects shortening of the atrial refractory period and of the wavelengths of reentry circuits. Such vagal over-stimulation creates a suitable environment for the occurrence and maintenance of AF.^34^ However, it is still unknown whether the correlation between AF and vagal stimulation affects the occurrence and maintenance of GERD. Postprandial paroxysm of AF is mediated by an efferent vagal nerve activity, which induces gastric juice secretion and esophageal sphincter relaxation, leading to acid reflux.^35^ In patients with either GERD or AF, vagal nerve over-stimulation has been observed. All of this points to a correlation between vagal nerve over-stimulation and GERD or AF.

What distinguishes the results of the present investigation is the significant correlation of GERD and AF, especially, presence of AF with increased risk of diagnosis of GERD. We found that the presence of previous AF was associated with an increased incidence of new-onset GERD (adjusted HR: 1.37, 95% CI: 1.16–1.57, *P* = 0.009). We considered local inflammation, anatomical and autonomic mechanisms as possible factors impacting our results. Although other factors (smoking, obesity, drug medication, etc.) would be risk factors for GERD occurrence in previous studies, there was no statistically significant risk factor influencing for occurrence of new-onset GERD, except presence of AF, in our study.

This study has a limitation. This study was a hospital-based, retrospective, case–control study. First, the diagnoses of AF and GERD are confirmed according to the *ICD-9* based on medical record review of subjects, there is a possibility that the exact prevalence of GERD may be underestimated because we could not fully investigate the patients without medical record or *ICD-9* codes. Second, it may be hard to draw conclusions from the comparison between AF group and the control group if the AF group was screened and the control group was not; further, if the AF group underwent more regular or more intensive overall medical evaluation than the control group, there may be a selection bias favoring a higher likelihood of identifying GERD in the AF group than in the control group.

In conclusion, the presence of AF appears to increase the incidence of GERD and may be considered a risk factor for the development of GERD. Considering the previous studies, we would recommend that all patients who develop AF should be screened for GERD or even treated empirically with PPI medications. Further large-scale prospective studies will be required in order to better establish the correlation of GERD with AF, to determine the actual GERD-AF mechanisms, and to assess whether they are dependent on a specific AF subtype.
